# Continuous Blood Pressure Estimation Based on Two-Domain Fusion Model

**DOI:** 10.1155/2018/1981627

**Published:** 2018-12-16

**Authors:** Qian Wang, Yajie Xu, Guoqiang Zeng, Mingshan Sun

**Affiliations:** ^1^College of Nuclear Technology and Automation Engineering, Chengdu University of Technology, Chengdu 610051, Sichuan, China; ^2^Medical Imaging Department, Suzhou Institute of Biomedical Engineering and Technology, Chinese Academy of Sciences, Suzhou 215163, Jiangsu, China

## Abstract

Blood pressure (BP) is one of the indispensable elements of physiological health characteristics and a significant indicator for predicting and diagnosing hypertension and cardiovascular diseases. This paper proposes a two-domain fusion model to estimate BP continuously from pulse wave acquired with a pressure sensor. *Method*. The optimal external pressure applied on the pressure sensor is first determined in order to capture pulse wave in the radial artery. The captured pulse wave is then processed in both the time and frequency domains via filtering and fast Fourier transform. Finally, a set of features are extracted from these two domains and input into a neural network along with blood pressure values measured by a commercial sphygmomanometer for training. The model is then tested on new data for accuracy evaluation. *Results*. The proposed two-domain fusion method achieved a high degree of accuracy in measuring blood pressure.

## 1. Introduction

According to the American Journal of Medicine, between 1990 and 2015, the rate of blood pressure (BP) greater than 140 mmHg rose from 17307/100000 to 20526/100000 and the associated mortality rate from 97.9/100000 to 106.3/100000 [1]. Admittedly, abnormal BP can cause a burden on the heart, which increases the risk of cardiovascular diseases [2, 3]. The traditional way of measuring blood pressure is to place a cuff on the upper arm and then detect changes in the pressure inside the cuff during inflation and deflation to obtain systolic blood pressure (SBP) and diastolic blood pressure (DBP). However, this method can hinder the circulation of blood and is not intended for continuous BP measurement.

In recent years, great progress has been made in noninvasive continuous BP measurement. A study found an inverse correlation between BP and the pulse transit time (PTT) [4, 5]. But this method requires an additional electrocardiogram (ECG) module, which brings complexity and inconvenience to portable blood pressure measurement. Wu et al. only used ECG signal to estimate BP. They took 6 features of the ECG and calculated the time between the relevant features. Two sets of ECG signal containing 4 time periods were combined and input into a neural network for training to get the BP. However, this model cannot simultaneously derive the BP and is significantly different compared with the target value [6]. Secondly, PTT has a significant correlation with systolic blood pressure but little relationship with diastolic blood pressure [7]. Xu et al. used PTT and photoplethysmogram (PPG) parameters to estimate blood pressure. They defined PTT as the different time between the R wave of ECG and the following pulse peak of PPG. They took the pulse wave amplitude, PTT, pulse wave period, and area as the parameters to send to a feed-forward back propagation neural network for training. Although the error could be within 5 mmHg, calibration was required before getting the output [8]. Luo et al. fabricated a low-power (3 nW) piezoresistive sensor (PS) device and measured BP using a single parameter PTT but also required clinical calibration of the initial values [9].

With the advancement of sensor technology, piezoelectric sensors nowadays become sensitive enough to reveal the pulse wave morphological features. However, during their use, piezoelectric sensors require an external force to balance the pressure between the probe and soft tissue [10]. In order to find the right amount of force applied to each individual, this paper proposes a method to use body mass index (BMI) to quantify the balance and find a suitable pressure for everyone. This will be explained in the third part of this paper.

Our goal is to develop a stable, efficient continuous blood pressure measurement system that requires no user calibration, to be used by patients with hypertension, cardiovascular, or other diseases for continuous BP monitoring. The paper is organized as follows: in Section 2, the background on propagation of pulse wave is given. In Section 3, a two-domain fusion model and its use with artificial neural networks (ANN) are elaborated.  Section 4 shows experimental results, and Section 5 provides a discussion and conclusion on this proposed method.

## 2. Background

**Figure 1:**
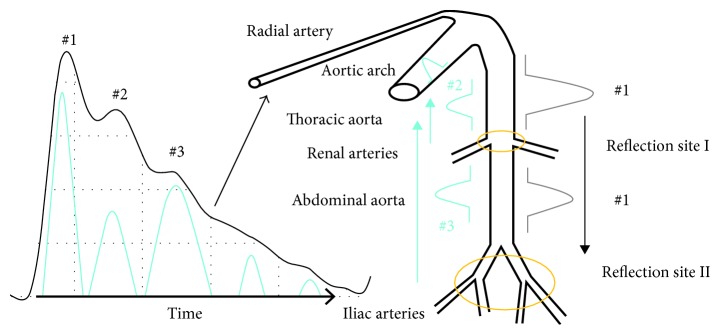
Relationship between pulse and BP.

Figure 1 shows pulse wave and blood propagation. When the cardiac ejection is completed, the central aorta forms a wave (#1), which is rebounded and generates a rebound wave (#2) when it propagates to the first reflection site (the arterial node between thoracic aorta and renal arteries). The rebound wave will be rebounded again, producing a wave (#3) when the wave (#1) gets to the second reflection site (the arterial node between abdominal aorta and iliac arteries). Finally, both of the rebound waves (#2 and #3) will superimpose on the main wave with a certain time delay and then propagate along the blood vessel to the radial artery or fingertip artery [11].

**Figure 2:**
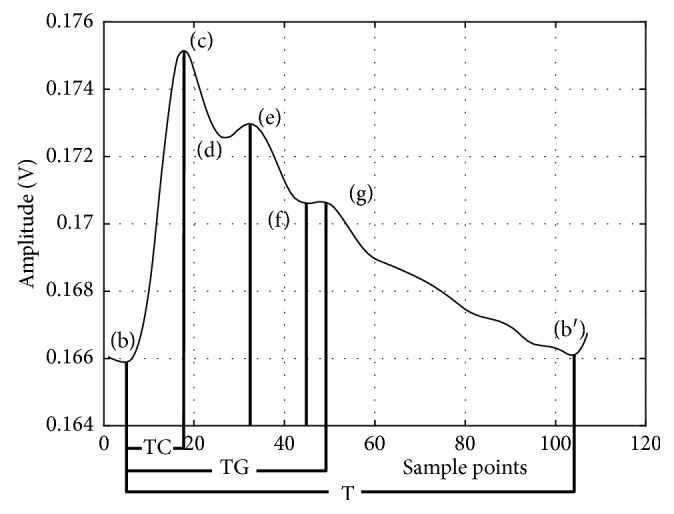
Standard pulse wave and characteristic points.

As shown in Figure 2, the features of the pulse wave mainly include (b)–(g). The (b)–(e) points correspond to the pulse wave's starting point, main peak, trough point, and the peak produced by the superposition between the first rebound wave (#2) and the main wave (#1). Points f and g are formed by the superposition between the second rebound wave (#3) and the main wave (#1). Note that in a young person with good blood vessel elasticity, the rebound wave can be weak and it can be submerged in the main wave and not visible in the waveform.

In order to accurately identify the characteristic points of the pulse wave, the pulse wave needs to be acquired with high signal-to-noise ratio (SNR) and without distortion. The detection of pulse wave feature points is shown below in the next section.

## 3. Methods

### 3.1. Pulse Wave Detection

There are two mainstream methods for pulse wave detection. One is photoplethysmogram (PPG), and the other is to apply a piezoelectric sensor (PS) in the radial artery. In PPG, light is emitted from a photodiode through a fingertip, a wrist, or the like, and the blood inside the artery absorbs the light and causes a change in the light intensity, so that a change in the amount of blood can be detected, therefore the pulse wave is obtained. The pulse wave signal in PPG is weak, and its SNR acquired by this method is generally not high. In addition, if a patient has arterial occlusion, tissue edema and blood clots, arrhythmia, or weak peripheral circulation, this method will show considerable deviations. While in PS, a pressure sensor is applied to the skin above the radial artery and records the pulse directly. The signal often has better SNR. Because one is an indirect measure and the other is a direct measure, the waveform characteristics collected by the two are different, and we have found that PS offers a lot of more details, thus more information, in its pulse waveform than in PPGs.

**Figure 3:**
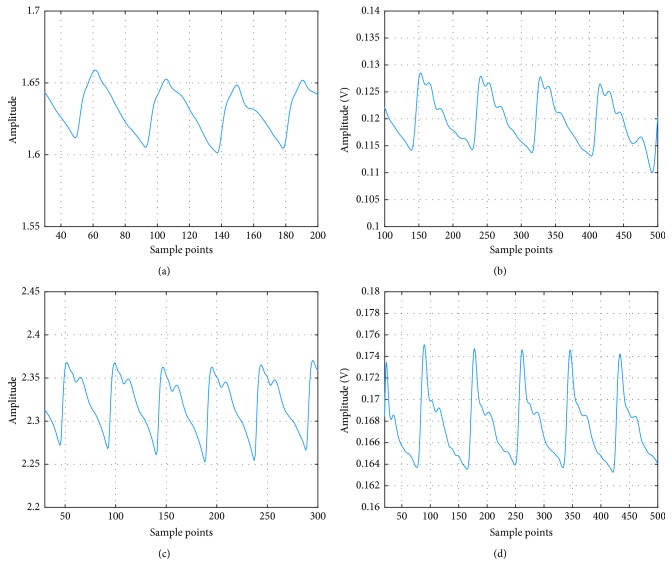
Pulse waves captured by PS and PPG. (a) PPG with arrhythmia. (b) PS with arrhythmia. (c) Normal PPG. (d) Normal PS.

Figures 3(a) and 3(b) are pulse waves from a 25-year-old with arrhythmia, collected by PPG and PS, respectively. In Figure 3(a), all the peaks except the main peak are all submerged due to the irregular heart beating. In comparison, there are three peaks in addition to the main peak clearly visible in Figure 3(b). Figures 3(c) and 3(d) are the normal PPG and PS waveform from a 26-year-old female. It can be seen that although the PPG can detect the pulse wave (note the 2nd peak is submerged), the ratio between the 3rd peak and the main peak is bigger than expected. The PS on the other hand has a better representation for the true pulse wave. Therefore, we will use PS as the method of pulse wave measurement for our study.

### 3.2. Optimal Pressure for Piezoresistive Sensor

As for the pressure sensor for blood waveform measurement, we choose Honeywell 1865 Series (Honeywell, Fort Mill, USA). It is a piezoresistive sensor that employs a solid-state piezopressure transducer mounted in a plastic package and offers high resolution using its Wheatstone bridge strain gauge design. It is designed to accurately collect tiny biological signals with good linearity, high test accuracy, and fast response. It is used here to accurately detect the pressure change caused by pulsation.

The sensor is applied on top of the radial artery with a certain external pressure to obtain the pulse wave by detecting the beat of the superficial artery on the surface of the skin. In actual measurements, the pressure applied to a same person can be different at different times, resulting in a difference in waveforms for the same individual and possibly errors in BP estimation. We thus need to find a constant and optimal pressure applied onto the sensor for each individual. Unfortunately, the research on this problem is still relatively muted. We will thus address this issue first before we move onto our two-domain fusion model.

Since the external force applied to the sensor is mainly to counter the pressure from the elasticity of the soft tissue, we hypothesize that the optimal pressure for an individual is dependent on the individual's physical measures such as height or obesity. We have thus done a lot of experiments to show this indeed is the case. When a force is applied to the detection unit and varied from small to large, the soft tissue under the sensor probe forms a force field. If the pressure of the sensor probe and the arterial wall of the radial artery are in close equilibrium, the shape and features of the pulse wave are the most conspicuous. Correspondingly, we define the pressure that produces the most detail of the pulse wave as the optimal pressure (OP) for capturing pulse wave. Admittedly, if you want to get different OP for different people, you need to rely on some indicator to quantify the soft tissue thickness above the radial artery. For this reason, we select the body mass index (BMI).

**Figure 4:**
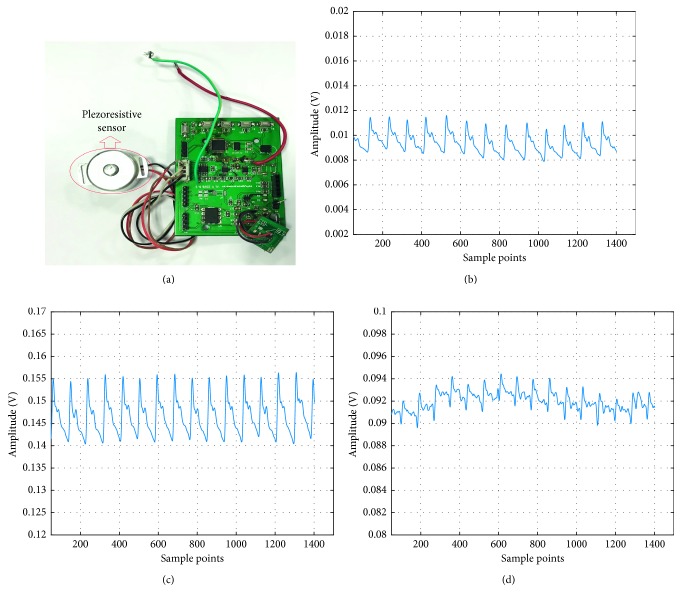
Pulse wave with different pressure. (a) Piezoresistive sensor. (b) Pulse wave at 2.3 N. (c) Pulse wave at 3.8 N. (d) Pulse wave at 14 N.

Figure 4(a) shows the piezoresistive sensor used in this article. It is equipped with a homemade casing and probe (the contact point or the tip) and signal processing board. Figures 4(b)–4(d) are pulse waves at the pressures 2.3 N, 3.8 N, and 14 N, respectively. At 2.3 N, the amplitude of the pulse wave is small, and the features are not clear. On the contrary, the pulse and its features are obvious at 3.8 N. Then at 14 N, the pulse wave is distorted with severe baseline shift and waveform overlapping. In order to get the OP, we collected pulse waves for each person at different external pressures. The steps are as follows:

**Step 1 .**

Find the place where the radial artery beats the most. Place the sensor probe on it.

**Step 2 .**

Place a piezoelectric film (Tekscan A201, Boston, USA) between the probe and the skin, adjust the wrist band so the average pressure reading is 2 N, and store 20 sets of pressure value in 500 ms cycles. Then, withdraw the piezoelectric film and measure the pulse wave at this time.

**Step 3 .**

Reinsert the piezoelectric film at the end of each measurement. Increase the pressure by 0.5 N and repeat step 2. Stop when the pressure reaches 14 N.

**Step 4 .**

Find a set of pulse waves with ideal amplitude and features and use median filter to fit the baseline of the corresponding pressure measured by the film.

**Step 5 .**

Find the value with the largest difference between the pressure value and the baseline and record the value of the baseline at this time.

**Step 6 .**

Repeat the above steps for 30 people and then use the BMI and the baseline values obtained in step 5 to perform a 3rd order polynomial fit to get the OP curve, where BMI is determined by the following equation:

(1) $$\begin{array}{c} \text{BMI}=\frac{\text{weight}\left(\text{Kg}\right)}{\text{height}{\left(\text{m}\right)}^{2}}. \end{array}$$

**Figure 5:**
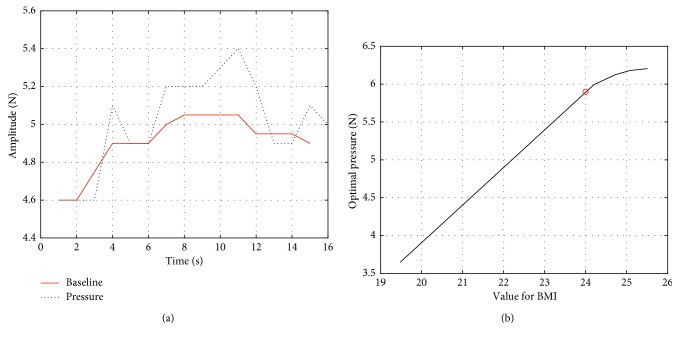
The pressure baseline and OP vs. BMI curve. (a) Baseline of pressure fitted with median filter. (b) Third-order polynomial fitting for optimal pressure of capturing pulse wave.

In Figure 5(b), when the BMI is less than 24, the OP curve is basically linear. It exhibits a saturation trend when the BMI goes beyond 24. Admittedly, the BMI of more than 24 is considered overweight in China [12]. So, we can come to a conclusion: when the BMI is lower than 24, the OP curve is linear with BMI. The OP is then determined by the following equation:

(2) $$\begin{array}{c} \text{OP}=-0.0114\;*\;{\text{BMI}}^{3}+0.7302\;*\;{\text{BMI}}^{2}-15.0889\;*\;\text{BMI}+104.4144. \end{array}$$

### 3.3. Two-Domain Fusion Model

After the pulse wave is acquired, we now move to the two-domain model. The analysis and decomposition of pulse waves so far in the literature were essentially in a single domain, that is, either in the time domain or in the frequency domain. Millasseau et al. have obtained a generalized transfer function (GTF) by the fast Fourier transform to analyze PPG and peripheral pressure pulse. They found that GTF is not only suitable for normotensive and hypertensive subjects, even adding few nitroglycerin (NTG, 500 mg sublingually) to the blood [13]. However, they only studied the peripheral pressure pulse analysis but nothing for blood pressure estimation. Xing et al. have decomposed the pulse wave in the frequency domain and found that the blood pressure has a high correlation with the amplitude of the fast Fourier transform and that the phase also has a good correlation in the low frequency part [14].

**Figure 6:**
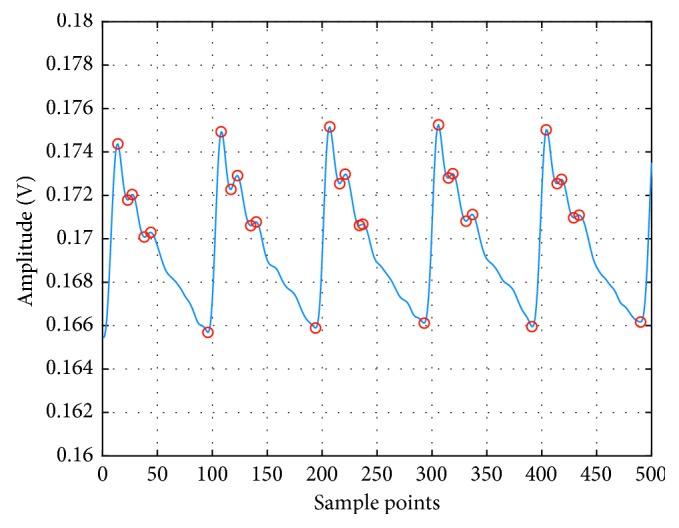
Feature points in the pulse wave in the time domain.

In the time domain, each morphological point of the pulse wave can be related to physiological features that are in turn related to the elasticity of the arteries and the ability of the heart to contract. One can derive blood pressure based on these characteristics. In Figure 6, the wave will be decomposed into 7 features in the time domain.

In order to prevent confusion in the identification of periodic features of several pulse waves, we extract features only in a single pulsation period and then identify other features in other pulsation period the same way.

**Figure 7:**
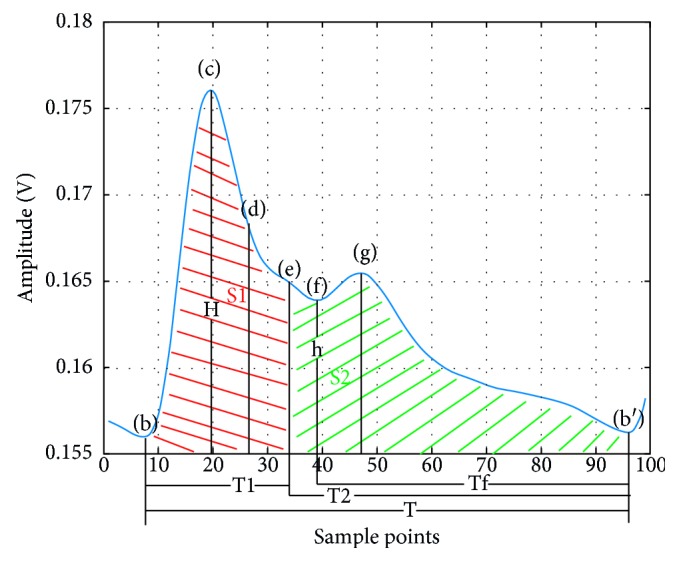
Illustration of time features.

At each systole, the amount of cardiac ejection is different. The blood is transmitted through the arterial wave and ultimately reflected in the amplitude of the systolic peak [15]. Peripheral resistance will change with the diameter of the artery and the viscosity of the blood, affecting the pulse wave [16]. If the elasticity of the distal end of the blood vessel is higher, the peak of the rebounding wave will be relatively lower, and the position superimposed on the main peak will change the ratio of the area between the systolic and diastolic points, which in turn affects the blood pressure [17, 18]. Furthermore, vascular elasticity has an influence on the PTT. The ratio of time between the main peak and other peaks can be different if arteriosclerosis, tissue edema, and clots are found out in the body [19]. The time domain focuses on features from a single beat, such as the ratio of each peak within one pulse wave, while the beat-to-beat variations will be taken into consideration in the frequency domain.  Figure 7 and Table 1 show the area of S1 (cardiac systole) and S2 (cardiac diastole) and other relevant parameters in the time domain.

**Figure 8:**
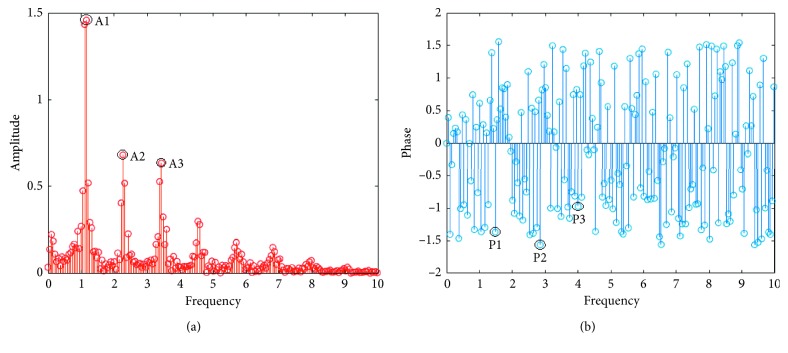
Illustration of frequency features. (a) FFT amplitude of pulse wave. (b) FFT phase of pulse wave.

In the frequency domain, as shown in Figure 8, there are substantial amplitude components at 0.3 Hz, 1.2 Hz, 2.4 Hz, 4.8 Hz, etc., after the fast Fourier transform (FFT) of the pulse wave, but the amplitude decays rapidly even almost submerge after 10 Hz. In this case, 0.3 Hz should be the frequency of breathing, 1.2 Hz is the heart rate, and the others are the multiple of the heart rate. It can be concluded that the main components of the pulse wave in the frequency domain are superimposed by the multiples or harmonics of the frequency of the heart rate and the frequency near the heart rate. The amplitude component corresponding to the frequency in the frequency domain of the pulse wave corresponds to the energy scale of each frequency. In order to improve accuracy and stability, this paper performs the FFT on five complete pulsation cycles for better frequency sampling. The features in the frequency domain are the amplitudes and corresponding phases of the first three characteristic peaks and are listed in Table 2. As for the other points, their amplitudes are smaller and may be interfered with the pulse wave noise.

The features from both domains are concatenated together and processed, combining the high precision of time domain and the stability of frequency domain into a better dual-domain fusion model. Experimental details are given below.

### 3.4. Implementation

**Figure 9:**
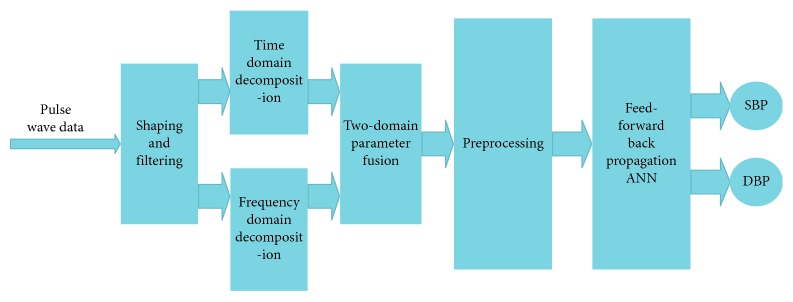
System structure.

After fitting and obtaining OP for each people according to their BMI, a group of subjects underwent measurement of pulse wave in the morning, afternoon, and evening for 3 days. In addition, each measurement is accompanied by BP obtained by a commercial medical upper-arm sphygmomanometer (Yuwell 670B, Jiangsu, China). Both the features of pulse wave and BP are fed into a neural network for supervised learning. The process flow is shown in Figure 9 and is described below.

**Step 7 .**

Collect real-time pulse wave data and then shape and filter to pulse wave.

**Step 8 .**

Decompose the pulse wave in the time and frequency domains.

**Step 9 .**

Reconstruct and fuse features in the time and frequency domains.

**Step 10 .**

Perform the mean shift and normalization with the recombined features.

**Step 11 .**

Send the processed features to the neural network to obtain SBP and DBP.

Note in the two-domain fusion model, the fusion is done in the input level of the neural network. We first exact features from the time and the frequency domains separately to form two vectors and then combine them into one data matrix to feed into the neural network for training. An additional label matrix records the true blood pressure measured by the commercial sphygmomanometer to serve as the training target. The neural network then iterates through the combined feature data, from which the weight corresponding to each feature is generated. The final trained model will contain coefficients for all time and frequency features, thus achieving the two-domain fusion.

**Figure 10:**
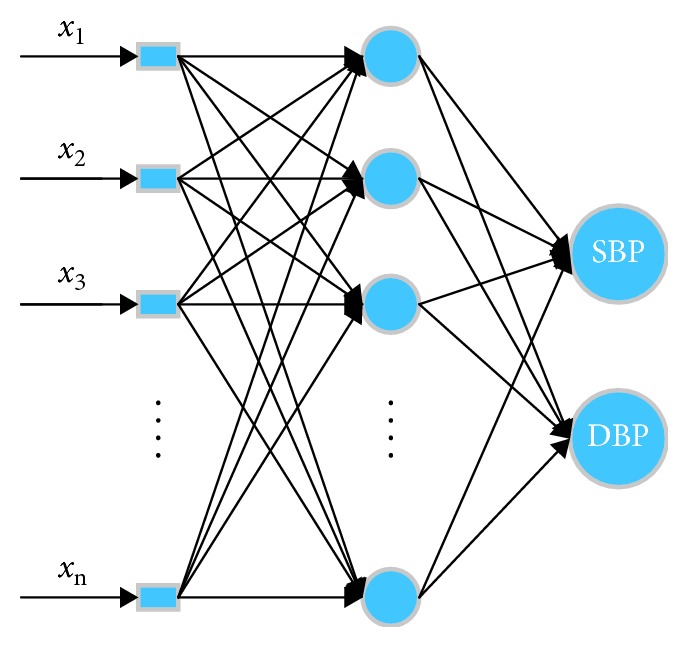
Structure of ANN.

In this paper, the quasi-Newton method is adopted for better convergence speed of the training. The neural network is a feed-forward back propagation ANN with 12 input points, 30 nodes in single hidden layer, and 2 output nodes. All 12 inputs of the network are the features extracted from the time domain and the frequency domain. The major superiorities of this type of network are as follows: (1) it is robust. Error in one input does little damage to the other inputs. (2) It can be proved mathematically that a three-layer network can approximate any continuous nonlinear relationship with arbitrary precision. (3) It has a strong self-adaptive learning ability and outputs adaptive learning contents to the weights of networks [8, 20]. In order to verify the training results, the data are randomly divided into 70%, 15%, and 15%, used for training, verification, and testing of the neural network, respectively. The structure of ANN is shown in Figure 10, where *x*_1_ to *x*_*n*_ are the spatial and frequency features listed in Tables 1 and 2.

In addition, the number of hidden layer nodes of the neural network is quantitatively analyzed to study the effectiveness after the number of hidden layer neurons has been increased. The results show that there is very little increase in accuracy when there are more than 30 hidden layer nodes. Furthermore, for the accuracy to increase by 0.01 mmHg, the training time goes up an order of magnitude by a network with a hidden layer of 50 neurons. Thus, we have chosen to use the aforementioned parameters for the ANN.

## 4. Experiment Results

**Figure 11:**
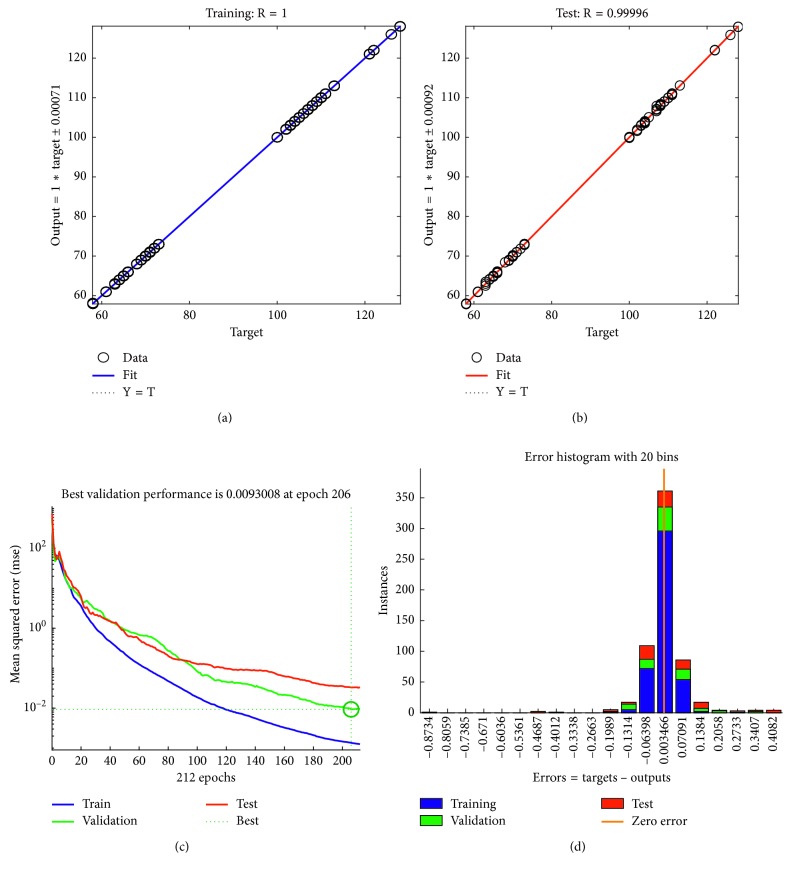
Two-domain model result. (a) Training error. (b) Testing error. (c) Iterations of ANN. (d) Two-domain error.

A total of 30 volunteers have participated in the experiment, and a total of 1231 pulse wave data were measured. In order to improve the accuracy and stability of blood pressure estimation, we collected the pulse waves in a single cycle. Besides, we built a threshold matrix to prevent any parameters, such as period and amplitude, exceeding the threshold range, upon which the data are considered abnormal and discarded. The training and fitting results of BP are shown in Figure 11.

The training automatically stopped after the about 200 iterations when the verification error became minimal. The total errors were within ±2 mmHg according to the error histogram (Figure 11(d)). In order to verify the performance of the model, we used the 15% data previously put aside and tested it. It is found that the output and target values show a highly linear correlation.

**Figure 12:**
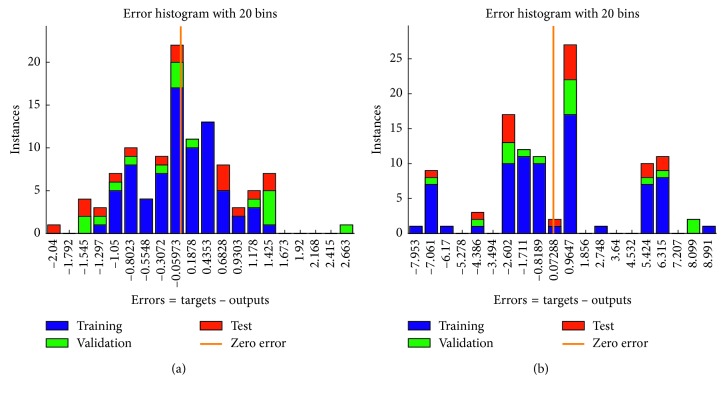
Errors of single-domain model. (a) Error of time domain. (b) Error of frequency.

In addition, we also compared our model results with that of the traditional single-domain model. The performance of the single-domain model is inferior to that of our two-domain model. As shown in Figure 12, the error is ±3 mmHg and ±9 mmHg in the time domain and the frequency domain, respectively.

## 5. Discussion and Conclusion

Experiments show that piezoresistive sensors can very well detect the features of the pulse wave under complex factors such as arrhythmia and are superior to PPG. There exists an optimal restraint pressure for the piezoresistive sensor, and it can be related back to the BMI for different people.

Considering the fact that the time domain model is unstable when measuring pulse wave data but has a high-accuracy contribution to BP and that the frequency domain model is stable in decomposing pulse waves, the two-domain model proposed in this paper not only retains the high accuracy of the time domain model but also integrates the stability of the frequency domain model. Experiments on the time and frequency domains and two domains combined were done and compared. The results show that the continuous blood pressure estimation based on the two-domain model can give BP with higher accuracy and stability than a single-domain model does.

There are limitations to this study, however. For example, the training and testing sample size is still small and not diverse enough. Subjects were not divided into training and testing groups, i.e., data from all subjects were mixed together and then divided for training and testing, which could introduce internal correlation. In addition, the features we choose to extract and use may not be the best to represent the underlying mechanisms of BP, and the number of features may not be the best. In the future, we will continue to optimize the feature selection in both the time and frequency domains and plan to enlarge the sample space and amend the method of data splitting for a more rigid evaluation study.

## Figures and Tables

**Table 1:** Features extracted in time domain.

Parameter	Definition
FC	The ratio between h and H
Sss	The ratio between S1 and S1 plus S2
Sds	The ratio between S2 and S1 plus S2
Tft	The ratio between Tf and T
Tst	The ratio between T1 and T
SL	The max slope between b and c

**Table 2:** Features extracted in frequency domain.

Parameter	Definition
A1	The amplitude of first peak
A2	The amplitude of second peak
A3	The amplitude of third peak
P1	The phase of first peak
P2	The phase of second peak
P3	The phase of third peak

## Data Availability

The datasets generated and analyzed during the current study are available from the corresponding author upon reasonable request, without breaching participant confidentiality.
